# Organochlorine Pesticides in Karst Soil: Levels, Distribution, and Source Diagnosis

**DOI:** 10.3390/ijerph182111589

**Published:** 2021-11-04

**Authors:** Wei Chen, Faming Zeng, Wei Liu, Jianwei Bu, Guofeng Hu, Songshi Xie, Hongyan Yao, Hong Zhou, Shihua Qi, Huanfang Huang

**Affiliations:** 1 State Key Laboratory of Biogeology and Environmental Geology, China University of Geosciences, Wuhan 430078, China; wei.chen@cug.edu.cn (W.C.); jwbu@cug.edu.cn (J.B.); shihuaqi@cug.edu.cn (S.Q.); 2 School of Environmental Studies, China University of Geosciences, Wuhan 430078, China; 3 Hubei Key Laboratory of Environmental Water Science in the Yangtze River Basin, China University of Geosciences, Wuhan 430078, China; 4 Hubei Provincial Engineering Research Center of Systematic Water Pollution Control, China University of Geosciences, Wuhan 430078, China; 5 State Key Laboratory of Organic Geochemistry, Guangzhou Institute of Geochemistry, Chinese Academy of Sciences, Guangzhou 510640, China; 6 Guangdong Province Key Laboratory of Environmental Protection, Chinese Academy of Sciences, Guangzhou 510640, China; 7 Resources Utilization, and Guangdong-Hong Kong-Macao Joint Laboratory for Environmental Pollution and Control, Chinese Academy of Sciences, Guangzhou 510640, China; 8 Institute of Geological Survey, China University of Geosciences, Wuhan 430074, China; wliu@cug.edu.cn (W.L.); zhouhong@cug.edu.cn (H.Z.); 9 Ecological Environment Monitoring Station, Ninth Division, Xinjiang Production and Construction Corps, Tacheng 834601, China; wowoyhy@163.com; 10 School of Environmental and Chemical Engineering, Foshan University, Foshan 528000, China; famingzeng@fosu.edu.cn; 11 China City Environment Protection Engineering Limited Company (CCEPC), Wuhan 430071, China; 54001@ccepc.com; 12 Shandong Institute of Geological Survey, Jinan 250013, China; sdsddy@163.com; 13 South China Institute of Environmental Sciences, MEE, Guangzhou 510535, China

**Keywords:** illegal use, non-point source pollution, agricultural use, veterinary use, Three Gorges

## Abstract

Excessive reclamation and improper use of agrochemicals in karst areas leads to serious non-point source pollution, which is of great concern and needs to be controlled, since contaminants can easily pollute groundwater due to the thin patchy soil and developed karst structures. The occurrences of organochlorine pesticides (OCPs) in karst soil were investigated by analyzing 25 OCPs in the karst soils near the Three Gorges Dam, China. The total concentrations of OCPs ranged 161–43,100 (6410 ± 9620) pg/g, with the most abundant compounds being *p*,*p*′-DDT and mirex. The concentration differences between the orchard and vegetable field and between upstream and downstream presented the influences of land-use type and water transport on the OCP spatial distributions. Composition analysis indicated the possible fresh inputs of lindane, technical DDT, aldrin, endrin, mirex, and methoxychlor. Their illegal uses implied an insufficient agrochemical management system in undeveloped karst areas. Principal component analysis with multiple linear regression analysis characterized the dominant sources from current agricultural use and current veterinary use in the study area. OCPs in the soils might not pose significant cancer risk for the residents, but they need to be controlled due to their illegal uses and bioaccumulation effect via the food chain.

## 1. Introduction

Organochlorine pesticides (OCPs), mainly including dichlorodiphenyltrichloroethane (DDT), hexachlorocyclohexane (HCH), chlordane, endosulfan, aldrin, and mirex, are a class of synthetic chlorine-contained pesticides. They can effectively cause insect spasms and eventually kill insects by opening the sodium ion channel in the neurons or nerve cells of insects, causing them to fire spontaneously [1]. Because of the excellent insecticidal effects, OCPs were widely and largely used in agriculture during the 1950s–1980s worldwide [2,3]. With the disclosure of the high toxicity on humans and wildlife, including cancers, allergies, and neurologic, reproductive and immune dysfunctions [4,5], most OCPs were listed in the Stockholm Convention and banned in over 130 countries since the 1970s. Nevertheless, the OCP pollution is still of concern: (1) because of the persistence, the high OCP residues are still detected in the soil, water, sediment, atmosphere, and biota [6,7,8]; (2) OCPs can undergo long-range transport within the atmosphere, water, and migrant birds, even to the places without any pesticide applications, leading to global pollution [9,10]; and (3) due to the lipophilicity, OCPs in the soil or water would accumulate in plants and livestock and eventually threaten human health via the food chain [11]. In addition, illegal uses of OCPs are still found in some countries and regions due to poor pesticide management. Recently, Khuman et al. (2020) reported the ongoing usage of technical HCH contradicting the ban in the agriculture sector on India’s southwest coast [12]. Fresh inputs were also observed for HCHs and heptachlor in soil and groundwater in the middle reaches of the Yangtze River Basin, China [13] and for DDTs in the soil from Mt. Shergyla, Tibetan Plateau, China [14]. These emphasize the need to continuously investigate the occurrence of OCPs in the environment and accordingly adjust policies for risk control.

With the area accounting for ca. 15% of the continental surface, karst is one of the most important landscapes on terrene and is home to a quarter of the global population [15]. Karst areas are mostly mountainous and dominated by agriculture economy. The soil resource is very precious in karst areas. On the one hand, as the carbonatite widely distributes, it is not easy to form soil in karst areas; the formation of one cm depth soil in karst areas might take 4000–8500 years [16]. On the other hand, as the transmissive network consisting of sinkholes, fissures, and conduits is well developed in the rainfall-dissolved carbonate bedrock [17], soil erosion is prevalent and severe in karst areas [18]. The soil in karst areas is generally thin, patchy, and fragile [19]. Nevertheless, farmers conduct agricultural activities, which is perhaps the most ubiquitous human activity on karst terranes, to feed themselves in this vulnerable soil layer. Agriculture has even been expanded to marginal soil on slopes and ridges due to the increase of population and the decline of land productivity [20], adversely affecting the ecology in karst areas, including the exacerbation of soil erosion, deforestation, and pollutions of fertilizers, pesticides, and agricultural wastes [21]. Among those, the non-point source pollution of agrochemicals in soil has raised great concerns because the soil contaminants pose adverse impacts on human health directly and via the food chain. To make matters worse, the thin patchy karst soil is not capable of buffering against pollutants; the soil contaminants can easily pollute the surface water and groundwater with rapid water runoff via highly permeable networks of fissures and conduits [22,23], leading to widespread pollution in the karst multimedia [24].

Many studies have focused on the OCP pollution in the karst water. The high OCP concentrations were reported in the surface river water (32.1–293, average 120 ng/L) [25], underground river water (2.58–320 ng/L) [25,26], spring water (0.30–32.2 ng/L) [27], and the sediment cores (0.85–63.1, average 8.11 ng/g) [28] in southwestern China, one of the largest karst areas in the world [29]. In the Yucatán karst area, México, severe OCP pollution (up to 1.36 × 10^7^ ng/L for heptachlor) was also reported in groundwater [30]. By contrast, there are much fewer investigations of OCPs in karst soil [31,32]. Because OCPs in the water enter via the soil [33], the OCP investigation in the karst soil is fundamental and crucial for diagnosing the source, implementing effective management practice, and developing a regulatory system for risk control.

To study the occurrence of OCPs in karst soil, we collected soil samples from the Yichang karst area near the Three Gorges Dam, central China (a typical karst mountainous area) and analyzed 25 OCP compounds to (1) investigate the levels, compositions, and spatial distributions of OCPs in the karst soil; (2) diagnose and quantify the OCP sources in the karst area; and (3) assess the carcinogenic risk posed by OCPs in the karst soil to residents.

## 2. Materials and Methods

### 2.1. Study Area and Sample Collection

The karst region in southwestern China (ca. 780,000 km^2^) [34], including Guizhou, western Guangxi, eastern Yunnan, southeastern Chongqing, southern Sichuan, western Hunan, and western Hubei, is the largest contiguous karst area with the most intense karst development in the world [35]. It is the most undeveloped remote mountainous area in China, and many counties therein are poverty-stricken. In these undeveloped areas, people rely on agricultural production but have relatively weak environmental awareness and low risk perception on handling agrochemicals.

**Figure 1:**
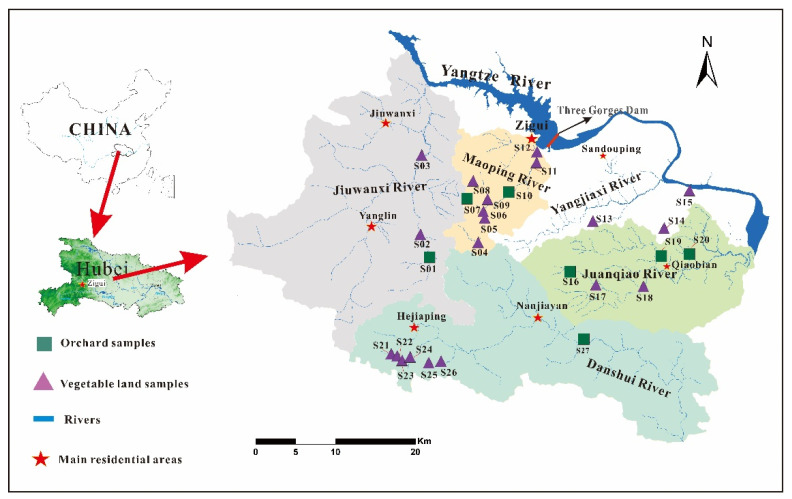
Location of the karst study area near Three Gorges Dam, China and the soil sampling sites.

The karst study area is in Yichang, western Hubei, with the area of approx. 2100 km^2^ (Figure 1). It mainly includes Zigui County (the first county closest to the Three Gorges Dam), in addition to part of Changyang County and Yiling and Dianjun Districts of Yichang City. The subtropical monsoon climate prevails in Yichang, with the average annual precipitation of 1216 mm and temperatures of 2–33 °C. The karst study area belongs to a karst trough zone (a typical landscape in central and southern China) [36] and has complex karst landforms consisting of middle-low mountains and deep ravines (40–2057 m a.s.l.). Numerous sinkholes, dolines, and grooves are developed on the up-platform, and large karst springs emerge in the deep valley. The soil layer in the study area is loose and highly uneven (0–4 m). Yellow soil, lime soil, and purple soil were dominant in this region. The pH in the soil ranges between 4.8–6.5, and the total organic carbon concentrations vary between 15.1–30.0 g/kg [37].

Although the mountainous karst area is not suitable for farming, cultivation is the most predominant human activity in this undeveloped area (the urbanization rate of Yichang is 44.4% [38]). The arable lands are scattered in big depressions on the up-platforms, slopes, and at the bottom of valleys. Farmers forge a living from cultivation on this thin soil overlying carbonate rocks by growing vegetables, flue-cured tobacco, tea, oranges, chestnuts, and other cash crops. Under the excessive reclamation, the soil layer in Zigui County had decreased by on about 3–5 cm depth per year [39] in the past and suffered severe agrochemical non-point source pollution [40].

The sampling campaign was conducted in October 2019 to avoid the impact of intensive agricultural activity. To collect soil samples that can represent the study area, sample sites were set in fields with relatively thick (>20 cm) and continuous soil layers (>20,000 m^2^). Twenty-seven surface soil samples (0–20 cm, ca. 1 kg for each) were collected in agricultural fields by clean stainless-steel shovels, of which seven samples were collected from orchards and twenty samples were collected from vegetable fields (Figure 1). After collection, soil samples were wrapped with pre-baked aluminum foil, sealed in PE zip bags and stored in a car refrigerator (4 °C, in the dark) during the sampling and transportation. Once delivered to the laboratory, soil samples were freeze-dried, preserved in the freezer (−20 °C, in the dark), and pretreated within seven days.

### 2.2. Sample Preparation and Analysis

Each dry soil sample (10 g dry weight) was spiked with 20 ng of 2,4,5,6-tetrachloro-*m*-xylene (TC*m*X) and decachlorobiphenyl (PCB209) as recovery surrogates [41,42], and then Soxhlet-extracted with 150 mL of dichloromethane (DCM) for 24 h. Before Soxhlet extraction, clean activated copper granules were added to the collection flask to remove the elemental sulfur from the extract during the extraction. After extraction, each extract was concentrated, solvent-exchanged to *n*-hexane, and reduced to 3 mL by a rotary evaporator (Heidolph G3, Schwabach, Germany). A neutral alumina/silica gel (*v*/*v*, 1:2) column was then used to purify each concentrated extract. Target OCP compounds were eluted with DCM/*n*-hexane (2:3, 30 mL). The eluate was then concentrated to 0.2 mL under a high-purified gentle nitrogen stream (99.999%) and stored in the sample freezer (−20 °C, in the dark). Before performing the instrumental analysis, each sample was spiked with 20 ng of pentachloronitrobenzene as the internal standard [43]. More details of the sample preparation for OCPs could be found in previous studies [44,45].

Target OCPs were analyzed with an HP7890A gas chromatograph-^63^Ni electron capture detector (GC-ECD, Agilent, Santa Clara, CA, USA) equipped with an HP-5MS column (30.0 m × 0.32 mm × 0.25 μm). According to a well-documented study in the same research group [44], the injector and detector temperatures were 290 and 300 °C, respectively, and the GC oven temperature program was set as: initially 100 °C for 1 min, 4 °C/min to 200 °C, 2 °C/min to 230 °C, and 8 °C/min to 280 °C for 15 min. The calibration curves used to quantify OCPs were built with six standards with increasing analytes concentrations (10, 20, 50, 100, 150, and 200 ng/mL) and 100 ng/mL pentachloronitrobenzene.

### 2.3. Quality Assurance and Quality Control (QA/QC)

Method blanks, parallel samples, blank solvent, QC standard samples, recovery surrogates, and internal standards were used to perform the QA and QC during the sample pretreatment and instrumental analysis. A method blank sample and a parallel sample were pretreated in each batch (16 samples) with the same procedures for the sample extraction and purification. For the instrumental QC, a blank solvent and a standard solution of OCPs were analyzed between every ten-sample analysis to check for interference/cross-contamination and instrument stability. Target compounds were undetectable in all blank samples and solvents. Relative standard deviation values were within 20% for the parallel samples and 10% for the QC standards. The recoveries of TC*m*X and PCB209 were 74.8 ± 17.3% and 86.9 ± 24.1%, respectively. Three times the signal-to-noise levels were used as the detection limits for target compounds. The method detection limits (MDLs) of OCPs were in the range of 1–20 pg/g (Table S1).

All data reported in this study were based on the GC-ECD analysis, but samples with high OCP concentrations were also confirmed with a gas chromatography–mass spectrometer (GC-MS, 6890N GC-5975MSD, Agilent, Santa Clara, CA, USA), and results showed the low relative percentile differences of OCP concentrations between GC-ECD and GC-MS analysis (<10%). Because of the higher MDLs of OCPs by GC-MS compared with GC-ECD, samples with relatively low OCP concentrations were not analyzed with GC-MS.

### 2.4. Data Analysis

According to the parent–daughter relationship and commercial formulas, twenty-five OCPs were divided into eight groups: HCB, HCHs (*α*-HCH, *β*-HCH, *γ*-HCH, and *δ*-HCH), DDTs (*p*,*p*′-DDT, *o*,*p*′-DDT, *p*,*p*′-DDE, *o*,*p*′-DDE, *p*,*p*′-DDD, *o*,*p*′-DDD), CHLs (*trans*-chlordane, *cis*-chlordane, heptachlor, and heptachlor epoxide), ENDOs (*α*-endosulfan, *β*-endosulfan, and endosulfan sulfate), DRINs (aldrin, dieldrin, endrin, endrin aldehyde, and endrin ketone), mirex, and methoxychlor. All concentrations in the soil were reported on a pg/g dry weight (dw) basis.

The Kruskal–Wallis test and Spearman correlation analysis were conducted to investigate the difference and correlation between OCP groups. The principal component analysis (PCA) with multiple linear regression analysis (MLRA) was used to identify and quantify the OCP sources in the karst soil. The methodology details were presented in Text S1. These statistical analyses were all performed by SPSS Statistics 25 (IBM, Chicago, IL, USA).

Human exposure to OCPs in soil is mainly via ingestion, dermal contact, and inhalation. In this study, the incremental lifetime risk of cancer (ICLR_total_) exposure to 25 OCPs in the soil via ingestion, dermal contact, and inhalation was assessed according to the US EPA Exposure Factors Handbook–1997 [46]. The ICLR_total_ was assessed for three population groups: children (3–10 years old), adolescents (11–18 years old), and adults (19–64 years old). Furthermore, risks of males and females were estimated separately. The parameters and calculation methods were analytically presented in Text S2.

## 3. Results and Discussion

### 3.1. General Comments on OCP Concentrations

**Figure 2:**
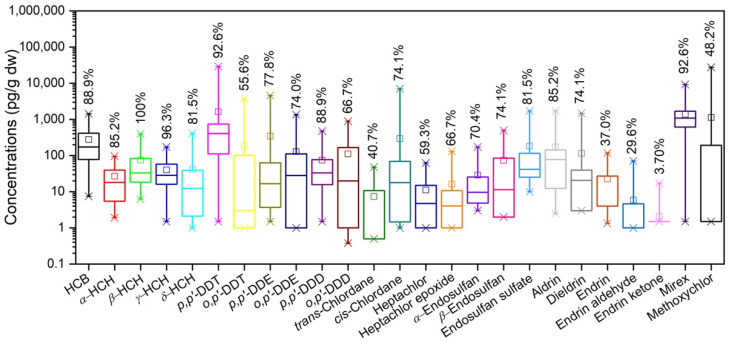
Concentrations and detection rates of individual OCPs in the study karst soil.

The total concentrations of OCPs (∑_25_OCPs) were in the range (mean ± standard deviation) of 161–43,100 (6410 ± 9620) pg/g (Table S1). The most abundant compounds were *p*,*p*′-DDT (1640 ± 5560 pg/g) and mirex (1410 ± 1720 pg/g) (Figure 2), accounting for the average 16.0% and 34.7% of the ∑_25_OCPs, respectively. With the detection rates of >85%, HCB, *α*-HCH, *β*-HCH, *γ*-HCH, *p*,*p*′-DDT, *p*,*p*′-DDD, aldrin, and mirex were prevalent in the study karst soil (Figure 2).

Compared with OCP concentrations in agricultural soils in other areas (Table S2), the HCB concentrations herein were within the ranges found in the Indus River Basin, Pakistan (400–1900 pg/g) [47] and Central Germany (570–3750 pg/g) [48], while these areas had higher ∑HCHs and ∑DDTs concentrations than ours. The ∑HCHs and ∑DDTs concentrations herein were also lower than those observed in the Pearl River Delta, southern China (<MDL–24,100 and 520–414,000 pg/g for ∑HCHs and ∑DDTs, respectively) [49], and the Sichuan Basin, southwestern China (69–3190 (avg. 1780) and 1870–25,200 (avg. 13,500) pg/g for ∑HCHs and ∑DDTs, respectively) [45].

### 3.2. Influence of Land-Use Type and Water Transport on the OCP Spatial Variation

**Figure 3:**
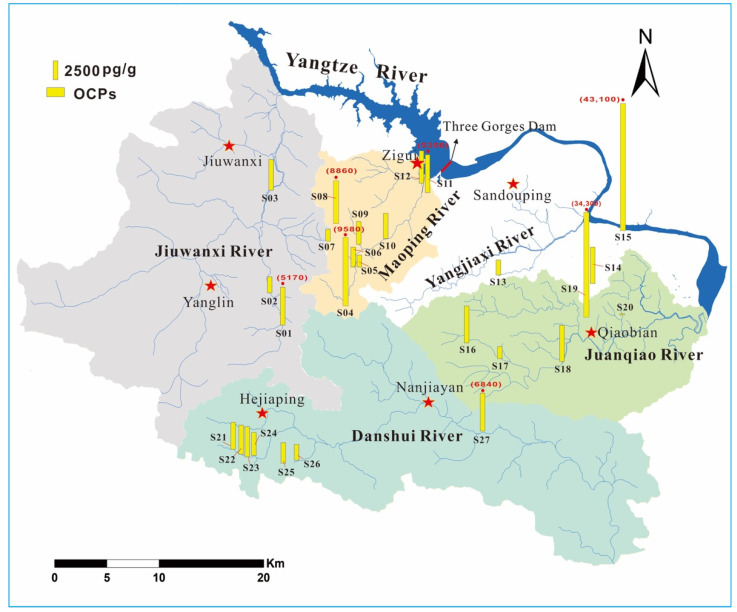
The spatial distribution of soil OCPs in different river basins.

Due to the hilly terrain and small farmland area, mechanized farming is not widespread in the study area. Individuals cultivated farmlands without unified management, which resulted in the high coefficients of spatial variations (CV) for ∑_25_OCPs (CV: 150%, Figure 3) and individual OCP compounds (CV: 94.8–520%, Figures S1–S3). The highest ∑_25_OCPs concentrations were found in Sites S15 and S19 from a vegetable field and an orange orchard, respectively (Figure 3), which might be attributed to the improper use of pesticides by farmers, or there might be agrochemical dumps in these sites. The Spearman correlation analysis (Table S3) showed that (1) HCB, HCHs, DDTs, CHLs, ENDOs, and DRINs were significantly correlated with each other; and (2) mirex and methoxychlor were not significantly correlated with any other OCP groups. These results indicated similar spatial distributions for OCP groups except for mirex and methoxychlor (Figures S1–S3).

The land-use type might affect the spatial distributions of OCPs in the karst area. The ∑_25_OCPs concentrations in the orchard (9000 ± 11,500 pg/g) were higher than those in the vegetable field (5510 ± 9020 pg/g). Specifically, the vegetable field had higher concentrations of HCB, HCHs, DDTs, CHLs, ENDOs, and DRINs compared with the orchard, while the concentrations of mirex and methoxychlor in the orchard were higher, although these comparisons were not significant (Kruskal–Wallis test, *p* > 0.05, Table S4). The higher concentrations of mirex and methoxychlor in the orchard soil might be due to their uses to treat tree mites, poultry, and livestock (and their sheds), as farmers keep poultry and livestock in orchards in the study area. Aside from the land-use type, the hydrogeological condition might also affect the spatial distributions of OCPs. Compared with the concentrations in upstream areas, higher concentrations were generally found in corresponding downstream sites for HCB, HCHs, DDTs, ENDOs, and DRINs in surface river basins (Figures S1 and S2). For example, Site S27 had higher ∑DDTs concentration than Sites S21–S26 in the Danshui River basin (Figure S1). The higher DDT concentrations in the downstream soil were also observed in the Minjiang River, Fujian, China [50]. This might be attributed to the collection of contaminants via tributaries and surface runoff, and the more extensive cultivations in the downstream areas. Mirex and methoxychlor had opposite spatial distributions with other OCP groups, i.e., they had higher concentrations in upstream areas (Figure S3), suggesting that compared with the water transport, the land-use type affected the spatial distributions of mirex and methoxychlor more significantly.

### 3.3. Source Diagnosis for OCPs by Composition Analysis

#### 3.3.1. HCB

HCB accounted for avg. 7.84% of ∑_25_OCPs in the soil (Figure S4a). HCB was a pesticide used to treat seeds and control wheat bunt [51], and was banned in 2009 in China [52]. However, HCB may still be emitted during industrial manufacturing, as HCB is a material of fireworks, ammunition, and synthetic rubbers [51,53]. In addition, coal combustion, waste incineration, and fuel combustion may also release HCB [51,54]. With the high atmosphere transport potential, HCB might also come from the long-range atmosphere transport from other areas. The study area is a remote mountainous area without industries around; thus, the industrial emission was not the main source of HCB herein. The contribution of long-range transport might also be only marginally indicated due to the high spatial variation of HCB (CV: 114%). Therefore, the locatable agricultural use was deemed the primary source for HCB in the soil.

#### 3.3.2. HCHs

The ∑HCHs concentrations accounted for avg. 5.43% of ∑_25_OCPs in the soil (Figure S4a). As shown in Figure S4b, *β*-HCH was the most abundant compound among four HCH isomers (accounting for avg. 38.9% of ∑HCHs), followed by *γ*-HCH. HCHs were generally introduced into the environment via the agricultural uses of technical HCH and lindane. Sources of HCHs could be distinguished as technical HCH and lindane have different formulas: technical HCH generally contains *α*-HCH (60–70%), *β*-HCH (5–12%), *γ*-HCH (10–15%), *δ*-HCH (6–10%), and other isomers (3–4%), while lindane contains a high content of *γ*-HCH (> 90%) [44,45]. In the environment, both *α*-HCH and *γ*-HCH can degrade (*γ*-HCH is more easily degraded) to *β*-HCH, which is more stable than its parent HCH compounds [55,56]. Furthermore, *γ*-HCH might be converted or biodegraded to *α*-HCH via photoisomerization and biodegradation [57,58]. *α*-/*γ*-HCH values of <4, 4–7, and >7 could therefore indicate the current-use of lindane, the current-use of technical HCH, and historical use of technical HCH, respectively.

**Figure 4:**
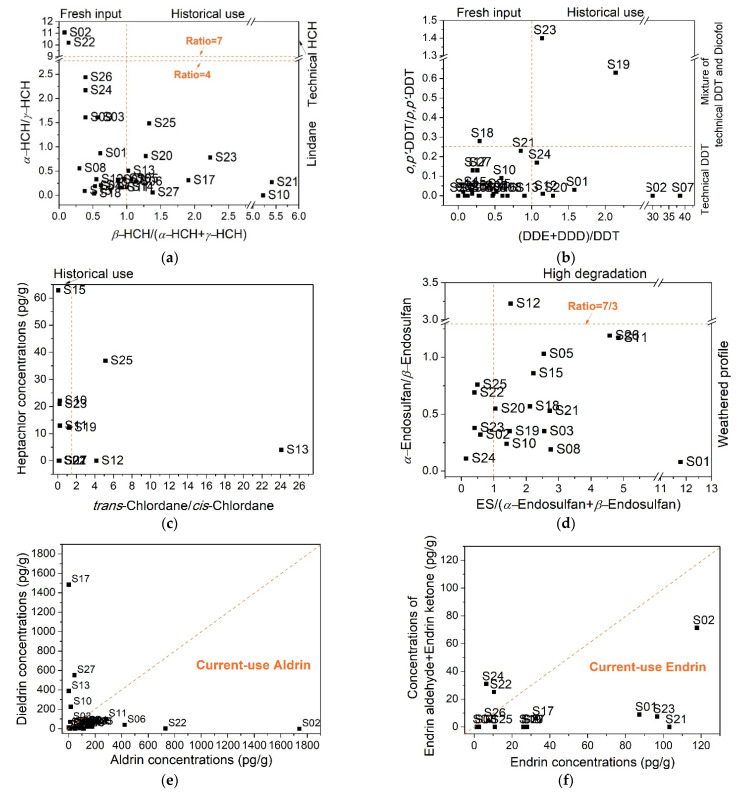
Isomeric and metabolic ratios for identifying the sources of HCH (**a**), DDT (**b**), chlordane (**c**), endosulfan (**d**), aldrin (**e**), and endrin (**f**) in the study karst soil. Some samples were not plotted because of undetectable target OCP compounds. Results showed the possible current uses of lindane, technical DDT, aldrin, and endrin in the soil.

The ratios of *β*-HCH/(*α*-HCH + *γ*-HCH) ranged from 0.09 to 5.41 (median: 0.87, Figure 4a) in the study karst soil. Only 40.7% of samples had ratios of >1, indicating that HCHs in the soil had not been highly degraded, i.e., there might be fresh input of HCHs herein. This was also supported by the low ratios of *α*-HCH/*γ*-HCH in the soil; the ratios of *α*-HCH/*γ*-HCH ranged from 0.04 to 11.1 (median: 0.34), with low ratios (<4) found in 92.6% samples, indicating the possible current use of lindane in the karst study area.

#### 3.3.3. DDTs

DDTs were one of the most important groups in the soil, accounting for avg. 28.2% of ∑_25_OCPs (Figure S4a). The most abundant compound was *p*,*p*′-DDT, accounting for avg. 54.9% of ∑DDTs (Figure S4c). Parent DDTs mainly degrade to DDE and DDD (*p*,*p*′- and *o*,*p*′-isomers included) under aerobic and anaerobic conditions, respectively. In this study, the values of DDE/DDD were in the range of 0–50.3, with low values (<1) found in 45.8% samples. This indicated the existence of anaerobic degradation of parent-DDTs in surface soil. Ratios of (DDE + DDD)/DDT ranged from 0.07 to 38.5, with low ratios (<1) found in 69.2% samples (Figure 4b), suggesting that DDTs in most sites were not highly degraded. Further analysis showed the low ratios (<0.25) of *o*,*p*′-DDT/*p*,*p*′-DDT in 88.0% of sites (Figure 4b), indicating the possible input of technical DDT (rather than dicofol) in our study area. This is unexpected because the agricultural use of technical DDT was banned in 1983, and its exception use for vector-control was also banned in 2009 in China [52]. The fresh input of technical DDT was also found in the air from a karst cave in Guilin [59], and in the soil from Chongqing, southwestern China [32]. Results herein implied the illegal use of technical DDT, and therefore insufficient pesticide management in the study area [60].

#### 3.3.4. CHLs

As a broad-spectrum insecticide on a range of crops, chlordane was used extensively to control termites. Technical chlordane in the international market contains 13% *trans*-chlordane, 11% *cis*-chlordane, and 5% heptachlor [61]. Of note, *trans*-chlordane is more prone to be photodegraded than *cis*-chlordane [62]. Thus, the *trans*-chlordane/*cis*-chlordane ratio is expected to be lower than 1.56 in the environment. In the study karst soil, the ∑CHLs concentrations accounted for avg. 5.04% of ∑_25_OCPs (Figure S4a). Here, *trans*-chlordane and *cis*-chlordane were rarely detected, with detection rates of 40.7% and 74.1%, respectively (Figure 2). Among the soil samples with both detectable *trans*-chlordane and *cis*-chlordane, *trans*-chlordane/*cis*-chlordane ratios varied from 0.06 to 24.1, with low ratios (<1) observed in 72.2% samples. This indicated the weathered chlordane profile in most soils. A very high *trans*-chlordane/*cis*-chlordane ratio (24.1) was found in Site S13. This might be attributed to the possible high aerobic degradation of *cis*-chlordane [63], rather than the use of heptachlor (commercial heptachlor contains 20–22% *trans*-chlordane), since the heptachlor concentration in Site S13 was low (4.02 pg/g, Figure 4c).

#### 3.3.5. ENDOs

Endosulfan was mainly used in cotton cultivation and was banned in China since 26 March 2019 [64]. The commercial endosulfan contains *α*-endosulfan and *β*-endosulfan in a ratio of 7:3 [65]. Both isomers can be degraded to endosulfan sulfate in the environment. In the study karst soil, endosulfan sulfate accounted for avg. 58.2% of ∑ENDOs (Figure S4e). The ratios of endosulfan sulfate/(*α*-endosulfan + *β*-endosulfan) ranged 0–34.3, with high values (>1) found in 73.9% samples (Figure 4d), indicating the high degradation of endosulfan in the soil. In samples with low ratio values (<1) of endosulfan sulfate/(*α*-endosulfan + *β*-endosulfan), the ratios of *α*-endosulfan/*β*-endosulfan ranged between 0.11–0.76 (Figure 4d), showing the weathered profile of endosulfan in the soil, as *α*-endosulfan was more prone to volatilize from the surface than *β*-endosulfan [66]. Considering the above, there might be no fresh input of endosulfan in the karst study area.

#### 3.3.6. DRINs

The concentrations of ∑DRINs merely accounted for avg. 6.88% of ∑_25_OCPs (Figure S4a), with the most abundant compound being aldrin (Figure S4f). Aldrin was used to kill termites, grasshoppers, and other insect pests. Its use had been banned since 2002 in China [67]. The aldrin concentration in the environment is generally low because aldrin can rapidly convert to dieldrin [68]. However, high ratios of aldrin/dieldrin (>1) were observed in 71.4% samples in this study, indicating the fresh input of aldrin. Endrin and its degradation products endrin aldehyde and endrin ketone were rarely detected in the soil (detection rates: <37.0%, Table S1). Among seven soil samples detected with at least one of these compounds, five samples have low ratios (<1) of (endrin aldehyde + endrin ketone)/endrin, indicating the possible current-use of endrin on a small scale.

#### 3.3.7. Mirex and Methoxychlor

Mirex is mainly used to combat ants and termites. It has also been used as a fire retardant in plastics, rubber, and electrical goods [68]. In China, the production, circulation, use, import, and export of Mirex had been banned since 2009 [52]. In the study karst soil, mirex was highly and frequently detected; it accounted for avg. 34.7% of ∑_25_OCPs (Figure S4a) with a detection rate of 92.6% (Table S1). In addition, mirex had high spatial variation (CV: 123%) with concentrations up to 9300 pg/g. The study area has suffered serious termite hazards. Based on the investigation and field survey of termite hazards in Zigui County conducted in January 2015, twenty-three out of thirty towns had serious hazards [69]. Using toxic pesticides is one of the methods used by residents to control termites. The high abundance and prevalence of mirex in the soil, accompanied by the serious termite hazards and termite control methods, might indicate the current use of mirex in the study area.

Methoxychlor was initially developed as a DDT replacement [70]. It was widely used in both agriculture (to treat field crops, vegetables, fruits, stored grains) and veterinary practices (to treat livestock, pets, homes, gardens) to combat biting flies, houseflies, and mosquito larvae [68]. Due to the acute toxicity, bioaccumulation, and endocrine disruption activity, methoxychlor was banned in 2003 in the USA [71] and is proposed for listing under the Stockholm Convention [70]. However, it is still used in some areas in China [72]. In the study karst soil, methoxychlor concentrations accounted for avg. 6.15% of ∑_25_OCPs (Figure S4a). Although methoxychlor was not prevalent in the soil (detection rate: 48.2%, Table S1), it had high spatial variation (CV: 470%) with concentrations up to 27,700 pg/g. This partly indicated the possible current input of methoxychlor herein.

### 3.4. Characteristics and Contributions of Sources

The OCP sources in the study karst soil were characterized by the PCA with MLRA, which were widely used in previous studies. For example, the sources of dicofol-type DDT, historical residues, and fresh technical DDT were drawn by the PCA + MLRA to explain 55%, 21%, and 17% of DDTs in the Pearl River Delta soil, southern China [49]. The PCA + MLRA indicated a greater contribution of the forest filter effect than the mountain cold trapping effect for the occurrence of polychlorinated biphenyls in the forest soil of Mt. Gongga, eastern Tibet [73].

**Figure 5:**
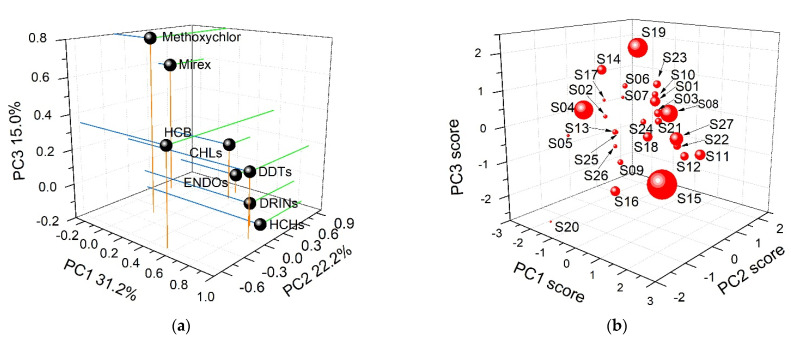

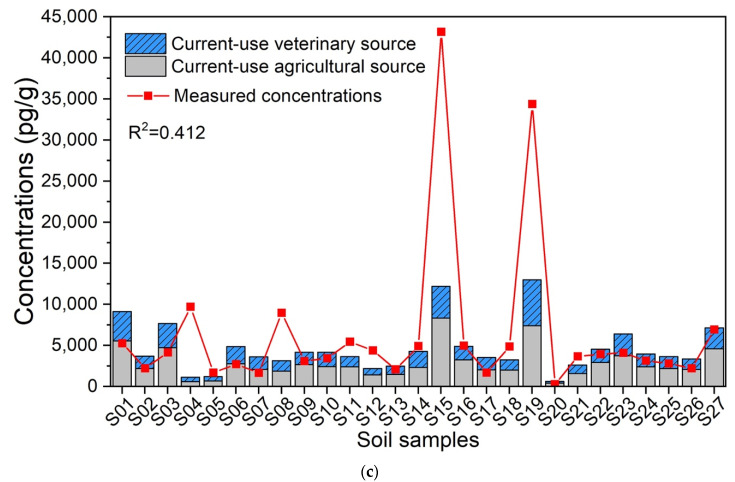
Loading profiles of PCs (**a**), factor scores of each soil samples (**b**), and contributions of current-use agriculture source and current-use veterinary source to the ∑_25_OCP concentrations in each soil samples (**c**), based on the PCA + MLRA analysis. PC1, PC2, and PC3 in (**a**) indicate the current agricultural use, historical agricultural use, and current veterinary use, respectively. The point sizes in (**b**) represent the concentration levels of ∑OCPs. The poor fits between modeled concentrations and measured concentrations in Sites S15 and S19 in (**c**) indicate the existence of other dominant pesticide sources (e.g., agrochemical waste dumps).

The log-transformed concentrations of HCB, HCHs, DDTs, CHLs, ENDOs, DRINs, mirex, and methoxychlor were used to perform the PCA (methodology details could be found in Text S1). Three PCs with eigenvalues greater than one were extracted, explaining 68.4% of the total variability. PC1 explained 31.2% of the total variability and had high loadings of HCHs, DDTs and DRINs, and medium loading of HCB; PC2 explained 22.2% and had high loadings of ENDOs and medium loading of CHLs; PC3 explained 15.0% and had high loading of methoxychlor and medium loading of mirex (Table S5 and Figure 5a). According to the source diagnosis results for different OCP groups in Section 3.3, PC1 and PC2 mainly contained compounds with possible fresh inputs and with weathered profiles, respectively. Therefore, PC1 and PC2 were identified as the current-use source and historical source, respectively. PC3 contained mirex and methoxychlor. There might also be current uses for these two compounds. However, they were clearly distinguished from other current-use pesticides (HCHs, DDTs, and DRINs in our study) (Figure 5a), indicating the different usages of mirex and methoxychlor. Mirex is mainly used against ants and termites. Methoxychlor was also widely used in veterinary practices to combat biting flies, houseflies, mosquito larvae, and cockroaches, aside from the agricultural use [68]. Therefore, PC3 might indicate the veterinary source of pesticides.

Soil samples were not regularly grouped (even for those located nearby) based on their PC scores (Figure 5b), showing the mixture OCP sources for most soils. For example, Sites S21–S26 were located nearby (Figure 1), but they were plotted separately in Figure 5b. This was attributed to the different pesticide uses due to individuals’ cultivation without unified management in the karst study area. Sites S15 and S19 were separated with predominant proportions of ∑DDTs (91.6%) and methoxychlor (80.8%), respectively, which were different from other samples.

Subsequently, the MLRA was performed to apportion the contributions of each source to the ∑_25_OCPs concentrations (see Text S1 for methodology details). The factor scores of PC1 and PC3 entered the regression equation, while PC2 was removed, suggesting the minor historical agricultural use for OCPs in the soil. The regression of PC1 and PC3 could merely explain 41.2% of the variation of log∑_25_OCPs (the dependent variable), indicated by the adjusted *R*^2^ value of 0.412 (Table S6, Figure S5). The fitted MLRA equation had statistical significance (ANOVA, *p* < 0.05, Table S7). The regression coefficients are shown in Table S8, and accordingly, the regression equation was:

(1) $$\log\sum_{25}\mathrm{OCPs}=0.566\times {\mathrm{PCS}}_{1}+0.368\times {\mathrm{PCS}}_{3}$$

where PCS_1_ and PCS_3_ were the factor scores of PC1 and PC3, respectively.

The contributions of PC1 and PC3 to the ∑_25_OCPs concentrations for each soil sample were then calculated, and are shown in Figure 5c. The MLRA could not exactly model the high ∑_25_OCPs concentrations in Sites S15 and S19, indicating that there might be other dominant sources in these sites, such as agrochemical waste dumps. The current agricultural use was dominant for the occurrence of OCPs herein, explaining 53.0–68.2% (average 60.5%) of ∑_25_OCPs. With the percent contributions of 31.8–47.0%, the current use of pesticides in veterinary practices should also be of concern.

### 3.5. Risk Assessment

The Soil Environmental Quality–Risk Control Standard for Soil Contamination of Agricultural Land (GB 15618-2018) from the China and Soil Remediation Circular 2009, from the Netherlands, was consulted to indicate the OCP pollution level in the soil. In the karst study area, the concentrations of ∑HCHs and ∑DDTs were lower than the Chinese risk screening values (both 1.0 × 10^5^ pg/g) for soil contamination of agricultural land [74]. Besides, all OCP concentrations were lower than the soil remediation intervention values for HCHs (1.2 × 10^6^–1.70 × 10^7^ pg/g), DDTs (1.7 × 10^6^–3.40 × 10^6^ pg/g), aldrin (3.2 × 10^5^ pg/g), and chlordane, endosulfan, and heptachlor epoxide (4.0 × 10^6^ pg/g) (Table S1) [75], indicating that the functional properties of soil in the karst study area for human, plant and animal life are not seriously impaired or threatened.

The incremental cancer risks calculated for children, adolescence, and adults were in the ranges of 9.52 × 10^−11^–1.46 × 10^−8^, 8.76 × 10^−11^–1.14 × 10^−8^, and 1.31 × 10^−10^–2.01 × 10^−8^, respectively (calculation details were presented in Text S2). The risk for adults was the highest among the three groups. In each group, males’ risk was slightly lower than that for females because of males’ higher body weight (Table S9). A risk lower than 10^−6^ was considered acceptable [76,77]. Therefore, OCPs in the soil would not pose a significant risk to residents. Nevertheless, the persistence and lipophilic affinity of OCPs would result in the bioaccumulation and biomagnification of these substances in crops and livestock, and they might eventually threaten human health via the food chain. Besides, as discussed in Section 3.3, fresh inputs of OCPs were found in the karst study area. Therefore, the risk of OCPs in soil should not be ignored.

## 4. Conclusions

The soil in karst areas has suffered severe non-point source pollution of agrochemicals due to the excessive reclamation and improper or illegal use of agrochemicals, which is especially crucial to control in karst areas since soil contaminants can easily enter surface water and groundwater owing to the thin patchy soil, fast water runoff, and developed karst fissures and caves. This study elaborated the occurrences of OCPs in karst soil by analyzing 25 OCPs in the soil from the Yichang karst area near the Three Gorges Dam, China. Results showed the total OCP concentrations of 161–43,100 pg/g dw. HCB, *α*-HCH, *β*-HCH, *γ*-HCH, *p*,*p*′-DDT, *p*,*p*′-DDD, aldrin, and mirex were frequently detected, of which *p*,*p*′-DDT and mirex were the most abundant compounds. The OCP spatial distributions were affected by the land-use type and water transport. The isomeric and metabolic ratios indicated the possible fresh inputs of lindane, technical DDT, aldrin, endrin, mirex, and methoxychlor. The PCA with MLRA analysis characterized the dominant sources of pesticides from current agricultural use and current veterinary use in the study karst soil.

The illegal uses and prevalence of OCPs implied the poor agrochemical management system, and farmers’ relatively weak environmental awareness and low-risk perception of handling agrochemicals, which might be attributed to poverty, low level of education, and lack of regulation in the agricultural sector. This social condition is a problem in the study area and in many remote karst areas worldwide, which is of great concern. Strict market regulation and professional training are urgently needed to prevent the illegal production, sale, and use of prohibited agrochemicals. Government and the public should recognize the ecological vulnerability in karst areas and take mitigation measures for sustainable development.

## Data Availability

The data that support the findings of this study are available from the corresponding author upon reasonable request.

## Supplementary Materials

The following are available online at https://www.mdpi.com/article/10.3390/ijerph182111589/s1, Text S1: Principle component analysis with multiple linear regression analysis; Text S2: Carcinogenic risk calculation; Figure S1: Spatial distributions of HCB, HCHs, DDTs, and DRINs in different river basins; Figure S2: Spatial distributions of CHLs and ENDOs in different river basins; Figure S3: Spatial distributions of mirex and methoxychlor in different river basins; Figure S4: The OCP compositions in the soil from the study karst area; Figure S5: The linear fit between the measured log∑25OCPs and modeled log∑25OCPs by the MLRA; Table S1: The OCP concentrations in the study karst soil; Table S2: Comparisons of OCPs in the agricultural soil between the study karst area and other areas around the world; Table S3: Spearman correlation coefficients between OCP groups; Table S4: OCP concentrations in different land use types; Table S5: Rotated component matrix; Table S6: The summary of the MLRA model for the OCP data in the study karst soil; Table S7: The ANOVA result for the MLRA; Table S8: The coefficients for the regression equation by MLRA; Table S9: The incremental lifetime cancer risk exposure to OCPs in soil from the study karst area.
