# Supplementary material for: Organochlorine Pesticides in Karst Soil: Levels, Distribution, and Source Diagnosis
Source: Int J Environ Res Public Health. 2021 Nov 4;18(21):11589. doi: 10.3390/ijerph182111589 (PMC8582917; doi:10.3390/ijerph182111589)
Supplement: Supplementary file 1 [file ijerph-18-11589-s001.zip › ijerph-1404247-supplementary.pdf]

Supplementary Materials for

Organochlorine pesticides in karst soil: levels, distribution, and source diagnosis

Wei Chen [1,2,3,4], Faming Zeng [5], Wei Liu [3], Jianwei Bu [1], Guofeng Hu [6], Songshi Xie [7], Hongyan Yao [4], Hong Zhou [3], Shihua Qi [1,2] and Huanfang Huang [2,8,*]

1. State Key Laboratory of Biogeology and Environmental Geology, and School of Environmental Studies, Hubei Key Laboratory of Environmental Water Science in the Yangtze River Basin, and Hubei Provincial Engineering Research Center of Systematic Water Pollution Control, China University of Geosciences, Wuhan 430078, China; wei.chen@cug.edu.cn (W.C.); jwbu@cug.edu.cn (J.B.); shihuaqi@cug.edu.cn (S.Q.)
2. State Key Laboratory of Organic Geochemistry, and Guangdong Province Key Laboratory of Environmental Protection and Resources Utilization, and Guangdong-Hong Kong-Macao Joint Laboratory for Environmental Pollution and Control, Guangzhou Institute of Geochemistry, Chinese Academy of Sciences, Guangzhou 510640, China
3. Institute of Geological Survey, China University of Geosciences, Wuhan 430074, China; wliu@cug.edu.cn (W.L.); zhouhong@cug.edu.cn (H.Z.)
4. Ecological Environment Monitoring Station, Ninth Division, Xinjiang Production and Construction Corps, Tacheng, Xinjiang 834601, China; wowoyhy@163.com
5. School of Environmental and Chemical Engineering, Foshan University, Foshan 528000, China; famingzeng@fosu.edu.cn
6. China City Environment Protection Engineering Limited Company (CCEPC), Wuhan 430071, China; 54001@ccepc.com
7. Shandong Institute of Geological Survey, Jinan 250013, China; sdsddy@163.com
8. South China Institute of Environmental Sciences, MEE, Guangzhou 510535, China

* Correspondence: hhuanfang@outlook.com

List of Texts

Text S1. Principle component analysis with multiple linear regression analysis

Page 3

Text S2. Carcinogenic risk calculation

Page 4-6

List of Tables

Table S1. The OCP concentrations (pg/g dw) in the study karst soil.
Page 7
Table S2. Comparisons of OCPs (pg/g dw) in the agricultural soil between the study karst area and other areas around the world.
Page 8
Table S3. Spearman correlation coefficients between OCP groups.
Page 9
Table S4. OCP concentrations in different land use types.
Page 9
Table S5. Rotated component matrix.
Page 10
Table S6. The summary of the MLRA model for the OCP data in the study karst soil.
Page 10
Table S7. The ANOVA result for the MLRA.
Page 10
Table S8. The coefficients for the regression equation by MLRA.
Page 10
Table S9. The incremental lifetime cancer risk exposure to OCPs in soil from the study karst area.
Page 11

List of Figures

Figure S1: Spatial distributions of HCB, HCHs, DDTs, and DRINs in different river basins.
Page 12

Figure S2: Spatial distributions of CHLs and ENDOs in different river basins.
Page 13

Figure S3: Spatial distributions of Mirex and Methoxychlor in different river basins.
Page 14

Figure S4. The OCP compositions in the soil from the study karst area.
Page 15
Figure S5. The linear fit between the measured log$\sum_{25}$OCPs and modeled log$\sum_{25}$OCPs by the MLRA.
Page 16

References

Page 17

Text S1. Principle component analysis with multiple linear regression analysis

The principal component analysis (PCA) with multiple linear regression analysis (MLRA) was widely used to identify and quantify the sources of organic contaminants in the environment. In this study, the PCA followed by MLRA was performed by IBM SPSS Statistics 25. The purpose of PCA is to extract a minimum number of factors (i.e., principal components, PCs) to represent the original OCP data's total variability. During the PCA, each original variable is orthogonal to all others to obtain the smallest possible covariance. Several PCs are

extracted to represent the weighted linear combinations of the original variables. Among them, PCs with eigenvalues greater than one are used to indicate the sources of OCPs. According to the source compositions, the source represented by PCs can be identified by evaluating the factor loading profiles of PCs. It is important to note that the OCP data used in the PCA must meet two assumptions: (1) the original variables are continuous, and (2) variables linearly correlate with each other. The adequacy of the original OCP data can be assessed by the Kaiser-Meyer-Olkin (KMO) and Bartlett's Test. The KMO value generally varies between 0 and 1, and the value higher than 0.6 indicates that the data is adequate and can be used to perform PCA. In the Bartlett's Test, the null hypothesis is that the original data's correlation matrix is perfect. There are no correlations between variables in this perfect matrix, and the original variables cannot be reduced to a few components, i.e., there is no need to extract PCs. Thus, in order to perform PCA, the result of Bartlett's Test for the original OCP data should reject the null hypothesis, i.e., the p-value of Bartlett's Test should be lower than 0.05. In this study, with the KMO value of 0.63 and the p-value of Bartlett's Test lower than 0.05 (Table S0-1), the original OCP data was adequate and can be used to perform the PCA.

After the source identification, the MLRA (stepwise) was performed to apportion the contributions of different sources to the $\sum_{25}$OCPs concentration for a given soil using the factor scores of PCs as the independent variables and the $\sum_{25}$OCPs concentration as the dependent variable. In this case, the independent variables' nonlinearities are ensured by using PCs' factor scores as the independent variables. A variable can only enter the regression equation during the stepwise procedure if it significantly increases the correlation (a default significance of 0.05 was used). The existing variable can only stay in the equation if its significance was higher than 0.10 [1]. The regression coefficients in the equation indicate the percent contributions of different sources.

Table S0-1. Results of Kaiser-Meyer-Olkin and Bartlett's Test for the original OCP data.

Kaiser-Meyer-Olkin measure of sampling adequacy.		0.63
Bartlett's Test of Sphericity	Approx. Chi-Square	51.881
	df	28
	Sig.	0.004

Text S2. Carcinogenic risk calculation

People exposure to OCPs in soil mainly via ingestion, dermal contact, and inhalation. According to the US EPA Exposure Factors Handbook – 1997 [2], the incremental lifetime risk of cancer (ICLR) for human exposure to chemical i via three ways were estimated as followed [3]:

$$\mathrm{ICLR}_{\mathrm{ingestion}\text{-}i} = \frac{\mathrm{C_{soil}} \times \mathrm{IR_{ingestion}} \times \mathrm{EF} \times \mathrm{ED}}{\mathrm{BW} \times \mathrm{AT}} \times \mathrm{CF} \times \mathrm{SF} \quad (1)$$

$$ICLR_{\text{dermal contact-}i} = \frac{C_{\text{soil}} \times SA \times AF \times ABS \times EF \times ED}{BW \times AT} \times CF \times SF \quad (2)$$

$$ICLR_{\text{inhalation-}i} = \frac{C_{\text{soil}} \times IR_{\text{inhalation}} \times EF \times ED \times \frac{1}{PEF}}{BW \times AT} \times SF \quad (3)$$

Where C_{soil} is the contaminant concentration in the soil (mg/kg), $IR_{ingestion}$ is the ingestion rate of soil (mg/day), EF is the exposure frequency to contaminated soil (days/year), ED is the exposure duration to contaminated soil (years), CF is the conversion factor (1×10^{-6} kg/mg), BW is body weight (kg), AT is the average lifetime (days), SF is the carcinogenic slope factor (chemical specific, 1/(mg/kg/day)), SA is skin surface area exposure to contaminants (cm^2), AF is the skin adherence factor for soil (mg/cm), ABS is the dermal absorption factor from soil (chemical specific), $IR_{inhalation}$ is the inhalation rate (m^3/day), and PEF is the particulate emission factor (1.36×10^9 m^3/kg).

The risk exposure to 25 OCP compounds through all exposure pathways ($ICLR_{total}$) was then calculated as:

$$ICLR_{\text{total}} = \sum_{i=1}^{25} \left(ICLR_{\text{ingestion-}i} + ICLR_{\text{dermal contact-}i} + ICLR_{\text{inhalation-}i} \right) \quad (4)$$

In this study, $ICLR_{total}$ in the soil was assessed for three population groups: children (3 – 10 years old), adolescents (11 – 18 years old), and adults (19 – 64 years old). Furthermore, risks of males and females were estimated separately in each group. Values of SF and ABS for each OCP compound were presented in Table S0-2, and other parameters used for ICLR calculations in this study were showed in Table S0-3.

Table S0-2. Slope factors (SF, 1/(mg/kg/day)) and dermal absorption factors (ABS, unitless) for target OCP compounds.

Compound	SF[a]	ABS[b]
HCB	1.6	
α-HCH	6.3	0.1
β-HCH	1.8	0.1
γ-HCH	1.3	0.04
δ-HCH		
p,p'-DDT	0.34	0.03
o,p'-DDT	0.34	**0.03**[c]
p,p'-DDE	0.34	
o,p'-DDE	0.34	
p,p'-DDD	0.24	0.1
o,p'-DDD	0.24	**0.1**
trans-Chlordane	0.35	0.04
cis-Chlordane	0.35	**0.04**

Heptachlor	4.5	
Heptachlor-epoxide	9.1	
α-Endosulfan		
β-Endosulfan		
Endosulfan sulfate		0.1
Aldrin	17	
Dieldrin		0.1
Endrin		0.1
Endrin aldehyde		
Endrin ketone		
Mirex		
Methoxychlor		0.1

[a]Data from U.S. EPA (http://www.popstoolkit.com/tools/HHRA/SF_USEPA.aspx)

[b]Data from Regional Screening Level (RSL) Summary Table [4]

[c]Bold values came from the values for their corresponding isomers.

Table S0-3. Parameters used for the assessment of the incremental lifetime cancer risk.

Parameters	Unit	Children (3-10 years old)		Adolescence (11-18 years old)		Adults (19-64 years old)		References
		Male	Female	Male	Female	Male	Female	
Body weight (BW)[a]	kg	34.0	32.7	51.0	47.9	68.0	57.5	[5]
Ingestion rate ($IR_{Ingestion}$)	mg/day	200	200	100	100	100	100	[6]
Exposure frequency (EF)	days/year	350	350	350	350	350	350	[6]
Exposure duration (ED)	years	6	6	14	14	30	30	[7]
Lifetime (LT)	years	73.6	79.4	73.6	79.4	73.6	79.4	[8]
Average life span (AT)	days	LT×365	LT×365	LT×365	LT×365	LT×365	LT×365	[6]
Skin surface area (SA)	cm^2	2,800	2,800	2,800	2,800	5,700	5,700	[6]
Dermal surface factor (AF)	mg/cm	0.2	0.2	0.2	0.2	0.07	0.07	[6]
Inhalation rate ($IR_{Inhalation}$)	m^3/day	8.4	8.4	13.1	13.1	14.8	14.8	[9]
Particle emission factor (PEF)	m^3/kg	1.36×10^9	1.36×10^9	1.36×10^9	1.36×10^9	1.36×10^9	1.36×10^9	[6]

[a]Body weights were calculated based on the investigation data during 2006-2011.

Table S1. The OCP concentrations (pg/g dw) in the study karst soil.

Compounds	MDL	Range	Median	Mean±SD	Detection rate	References values	
						Dutch*	China**
HCB	15	<MDL-1,440	172	278±316	88.9%		
α-HCH	4	<MDL-95.8	18.3	26.8±26.1	85.2%	1.70×10^{7}	
β-HCH	5	6.31-398	33.4	75.6±96.6	100%	1.60×10^{6}	
γ-HCH	3	<MDL-174	28.4	40.1±38.0	96.3%	1.20×10^{6}	
δ-HCH	2	<MDL-414	12.5	41.3±85.4	81.5%		
p,p′-DDT	3	<MDL-29,200	406	1,640±5,560	92.6%	1.70×10^{6}[a]	
o,p′-DDT	2	<MDL-3,690	2.99	189±708	55.6%		
p,p′-DDE	3	<MDL-4,610	16.7	346±942	77.8%	2.30×10^{6}[b]	
o,p′-DDE	2	<MDL-1,350	28.2	128±288	74.1%		
p,p′-DDD	3	<MDL-479	33.5	74.1±115	88.9%	3.40×10^{6}[c]	
o,p′-DDD	2	<MDL-901	20.0	111±195	66.7%		
trans-Chlordane	1	<MDL-48.3	<MDL	7.09±11.5	40.7%	4.0×10^{6}	
cis-Chlordane	2	<MDL-7,060	17.9	296±1,350	74.1%		
Heptachlor	2	<MDL-62.9	4.77	10.9±14.7	59.3%	4.0×10^{6}	
Heptachlor-epoxide	2	<MDL-131	4.09	16.1±33.8	66.7%	4.0×10^{6}	
α-Endosulfan	6	<MDL-174	9.71	28.4±43.4	70.4%	4.0×10^{6}	
β-Endosulfan	4	<MDL-501	11.4	73.2±125	74.1%		
Endosulfan sulfate	20	<MDL-1,720	41.4	182±376	81.5%		
Aldrin	5	<MDL-1,740	78.2	172±350	85.2%	3.2×10^{5}	
Dieldrin	6	<MDL-1,490	20.6	116±302	74.1%		
Endrin	8	<MDL-118	1.34	20.6±35.9	37.0%		
Endrin aldehyde	2	<MDL-71.4	<MDL	5.22±14.4	29.6%		
Endrin ketone	3	<MDL-17.4	<MDL	0.64±3.34	3.70%		
Mirex	3	<MDL-9,300	1,090	1,410±1,720	92.6%		
Methoxychlor	3	<MDL-27,700	<MDL	1,130±5,310	48.2%		
∑HCHs		18.2-549	108	184±161	100%		1.0×10^{5}
∑DDTs		25.6-39,500	659	2,490±7,480	100%		1.0×10^{5}
∑CHLs		1.09-7,060	66.1	330±1,350	100%		
∑ENDOs		<MDL-2,400	81.7	284±507	96.3%		
∑DRINs		9.42-1,930	183	314±452	100%	4.0×10^{6}	
$\sum_{25}$OCPs		161-43,100	3,850	6,410±9,620	100%		

*Dutch intervention values for soil remediation [10].

**Data from State Administration for Market Regulation, Ministry of Ecology and Environment, PRC (2018).

[a]This value was for the sum of *p,p′*-DDT and *o,p′*-DDT.

[b]This value was for the sum of *p,p′*-DDE and *o,p′*-DDE.

[c]This value was for the sum of *p,p′*-DDD and *o,p′*-DDD.

Table S2. Comparisons of OCPs (pg/g dw) in the agricultural soil between the study karst area and other areas around the world.

Locations	HCB	∑HCHs	∑DDTs	∑CHLs	∑ENDOs	∑DRINs	Mirex	Methoxychlor	Years	References
Zigui karst area, Central China	<15-1,440 (278)[a]	18.2-549 (184)	25.6-39,500 (2,490)	1.09-7,060 (330)	<30-2,400 (284)	9.42-1,930 (314)	<3-9,300 (1,410)	<3-27,700 (1,130)	2019	This study
Tibet Plateau, West China	(22)	(393)	(1,050)	(179)	(146)		(23)		2010	[12]
Pearl River Delta, South China		<MDL[b]-24,100	520-414,000						2002	[13]
Sichuan Basin, Southwest China	240-421 (330)	369-3,190 (1,780)	1,870-25,200 (13,500)	0.48-5.71 (3.10)	4.83-78.3 (41.5)	<6.76		<0.87-3.56 (1.78)	2015	[14]
Indus River Basin, Pakistan	400-1,900	7,000-27,000	54,000-320,000	1,700-16,000	500-2,700				2013	[3]
Central Germany	570-3,750	460-11,500	23,700-173,000						1995-1996	[15]

[a]Concentration range (average)

[b]Method detection limit

Table S3. Spearman correlation coefficients between OCP groups.

	HCB	∑HCHs	∑DDTs	∑CHLs	∑ENDOs	∑DRINs	Mirex
∑HCHs	0.409*						
∑DDTs	0.437*	0.654**					
∑CHLs	-0.034	0.321	0.334				
∑ENDOs	-0.192	0.452*	0.392*	0.631**			
∑DRINs	0.256	0.615**	0.445*	0.360	0.360		
Mirex	-0.180	-0.215	-0.178	-0.277	0.070	-0.077	
Methoxychlor	0.108	-0.229	0.026	0.058	0.075	0.084	0.206

*Correlation is significant at the 0.05 level (2-tailed).

**Correlation is significant at the 0.01 level (2-tailed).

Table S4. OCP concentrations in different land-use types.

Groups	Orchard (*n*=7)			Vegetable land (*n*=20)			Asymp. Sig. of Kruskal-Wallis Test
	Range	Median	Mean±SD[a]	Range	Median	Mean±SD	
HCB	16.7-557	92.6	205±213	<MDL[b]-1,430	179	303±345	0.543
∑HCHs	20.0-260	154	148±100	18.2-549	108	196±177	0.825
∑DDTs	25.6-3,630	1,700	1,710±1,580	56.8-39,500	625	2,760±8,690	0.740
∑CHLs	1.09-201	32.7	63.4±69.1	6.56-7,060	72.0	423±1,560	0.347
∑ENDOs	11.5-1,010	102	260±357	<MDL-2,390	63.4	293±557	0.619
∑DRINs	9.42-623	183	214±209	12.1-1,930	180	349±510	0.782
Mirex	<MDL-9,300	1,290	2,400±3,160	<MDL-2,540	1,030	1,060±630	0.234
Methoxychlor	<MDL-27,700	<MDL	4,000±10,400	<MDL 1,310	4.27	123±294	0.905
$\sum_{25}$OCPs	161-34,300	4,760	9,000±11,500	1,560-43,100	3,310	5,510±9,020	0.203

[a]Standard derivation

[b]Method detection limit

Table S5. Rotated component matrix[a]

	PC1	PC2	PC3
HCB	**0.539**[b]	**-0.586**	0.341
HCHs	**0.825**	0.269	-0.183
DDTs	**0.860**	0.012	0.169
CHLs	0.323	**0.639**	0.142
ENDOs	0.295	**0.813**	-0.084
DRINs	**0.749**	0.236	-0.069

Mirex	-0.173	0.467	**0.591**
Methoxychlor	0.066	-0.127	**0.800**

[a]Rotation method was varimax with Kaiser normalization and rotation converged in 6 iterations.

[b]Bold values were the high and median loadings.

Table S6. The summary of the MLRA model for the OCP data in the study karst soil.

R	R Square	Adjusted R Square	Std. error of the estimate
0.675	0.456	0.412	0.75249437

Table S7. The ANOVA result for the MLRA.

	Sum of Squares	df	Mean Square	F	Sig.
Regression	11.844	2	5.922	10.458	0.001
Residual	14.156	25	0.566		
Total	26.000	27			

Table S8. The coefficients for the regression equation by MLRA

	Unstandardized Coefficients		Standardized Coefficients	t	Sig.	95.0% Confidence Interval for B	
	B	Std.	Beta			Lower Bound	Upper Bound
PCS_1	0.566	0.148	0.566	3.833	0.001	0.262	0.87
PCS_3	0.368	0.148	0.368	2.495	0.020	0.064	0.672

Table S9. The incremental lifetime cancer risk exposure to OCPs in the karst soil.

Soil samples	Children (3-10 years old)		Adolescence (11-18 years old)		Adults (19-64 years old)	
	Males	Females	Males	Females	Males	Females
S01	3.36×10^{-9}	3.23×10^{-9}	2.69×10^{-9}	2.65×10^{-9}	4.25×10^{-9}	4.66×10^{-9}
S02	1.48×10^{-9}	1.42×10^{-9}	1.20×10^{-9}	1.19×10^{-9}	1.89×10^{-9}	2.07×10^{-9}
S03	1.67×10^{-9}	1.61×10^{-9}	1.65×10^{-9}	1.63×10^{-9}	2.33×10^{-9}	2.56×10^{-9}

S04	1.71×10^{-10}	1.65×10^{-10}	1.42×10^{-10}	1.40×10^{-10}	2.20×10^{-10}	2.41×10^{-10}
S05	4.99×10^{-10}	4.81×10^{-10}	3.92×10^{-10}	3.87×10^{-10}	6.26×10^{-10}	6.87×10^{-10}
S06	1.79×10^{-9}	1.73×10^{-9}	1.44×10^{-9}	1.42×10^{-9}	2.27×10^{-9}	2.49×10^{-9}
S07	1.15×10^{-9}	1.11×10^{-9}	9.35×10^{-10}	9.23×10^{-10}	1.47×10^{-9}	1.61×10^{-9}
S08	1.89×10^{-9}	1.82×10^{-9}	1.62×10^{-9}	1.60×10^{-9}	2.46×10^{-9}	2.70×10^{-9}
S09	4.47×10^{-9}	4.30×10^{-9}	3.57×10^{-9}	3.52×10^{-9}	5.65×10^{-9}	6.19×10^{-9}
S10	1.36×10^{-9}	1.31×10^{-9}	1.11×10^{-9}	1.10×10^{-9}	1.74×10^{-9}	1.91×10^{-9}
S11	1.46×10^{-8}	1.41×10^{-8}	1.14×10^{-8}	1.13×10^{-8}	1.83×10^{-8}	2.01×10^{-8}
S12	1.30×10^{-9}	1.25×10^{-9}	1.11×10^{-9}	1.10×10^{-9}	1.69×10^{-9}	1.86×10^{-9}
S13	8.35×10^{-10}	8.05×10^{-10}	6.64×10^{-10}	6.55×10^{-10}	1.05×10^{-9}	1.16×10^{-9}
S14	4.72×10^{-10}	4.55×10^{-10}	4.30×10^{-10}	4.25×10^{-10}	6.33×10^{-10}	6.94×10^{-10}
S15	1.08×10^{-8}	1.04×10^{-8}	1.09×10^{-8}	1.07×10^{-8}	1.52×10^{-8}	1.66×10^{-8}
S16	1.53×10^{-9}	1.48×10^{-9}	1.67×10^{-9}	1.65×10^{-9}	2.25×10^{-9}	2.46×10^{-9}
S17	9.64×10^{-10}	9.29×10^{-10}	7.74×10^{-10}	7.64×10^{-10}	1.22×10^{-9}	1.34×10^{-9}
S18	2.05×10^{-9}	1.98×10^{-9}	1.85×10^{-9}	1.83×10^{-9}	2.74×10^{-9}	3.01×10^{-9}
S19	2.99×10^{-9}	2.88×10^{-9}	3.17×10^{-9}	3.13×10^{-9}	4.32×10^{-9}	4.73×10^{-9}
S20	9.87×10^{-11}	9.52×10^{-11}	8.82×10^{-11}	8.76×10^{-11}	1.31×10^{-10}	1.44×10^{-10}
S21	5.99×10^{-9}	5.77×10^{-9}	4.71×10^{-9}	4.65×10^{-9}	7.52×10^{-9}	8.25×10^{-9}
S22	1.60×10^{-9}	1.54×10^{-9}	1.45×10^{-9}	1.43×10^{-9}	2.14×10^{-9}	2.35×10^{-9}
S23	1.52×10^{-9}	1.46×10^{-9}	1.30×10^{-9}	1.29×10^{-9}	1.98×10^{-9}	2.17×10^{-9}
S24	1.59×10^{-9}	1.53×10^{-9}	1.29×10^{-9}	1.27×10^{-9}	2.02×10^{-9}	2.21×10^{-9}
S25	1.05×10^{-9}	1.01×10^{-9}	8.99×10^{-10}	8.88×10^{-10}	1.37×10^{-9}	1.50×10^{-9}
S26	9.73×10^{-10}	9.37×10^{-10}	8.29×10^{-10}	8.18×10^{-10}	1.27×10^{-9}	1.39×10^{-9}
S27	1.77×10^{-9}	1.70×10^{-9}	1.57×10^{-9}	1.55×10^{-9}	2.34×10^{-9}	2.57×10^{-9}

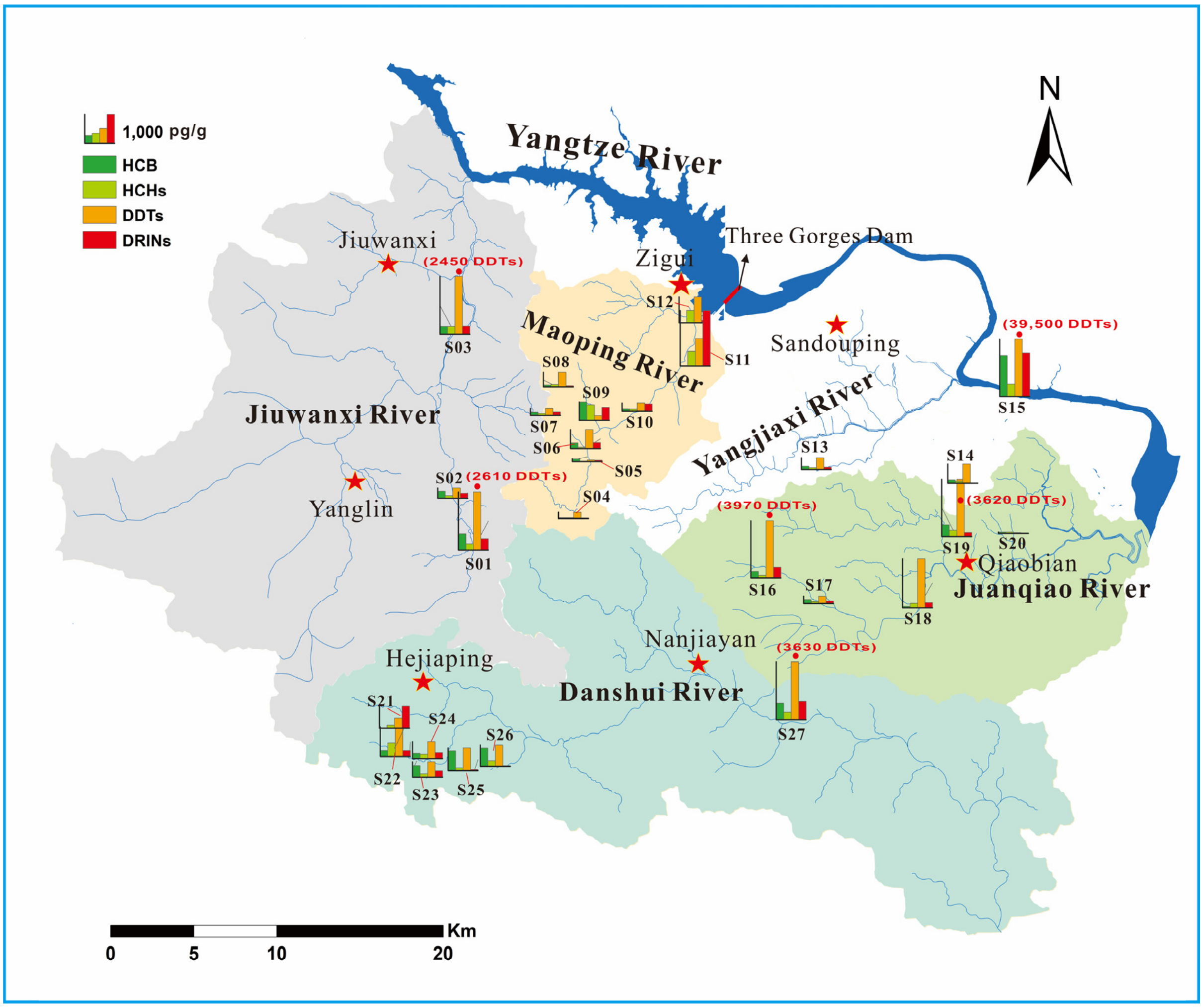

Figure S1. Spatial distributions of HCB, HCHs, DDTs, and DRINs in different river basins.

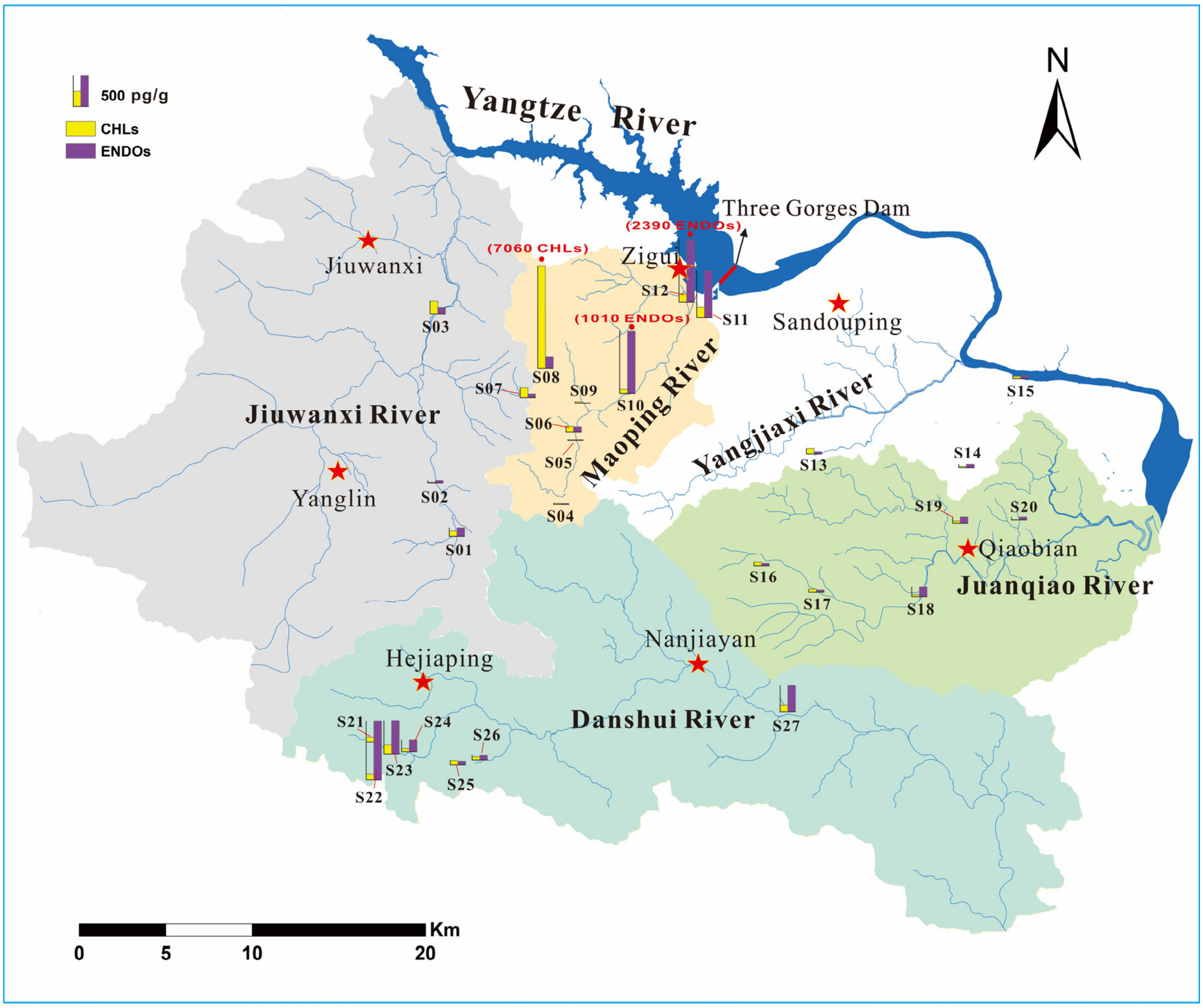

Figure S2. Spatial distributions of CHLs and ENDOs in different river basins.

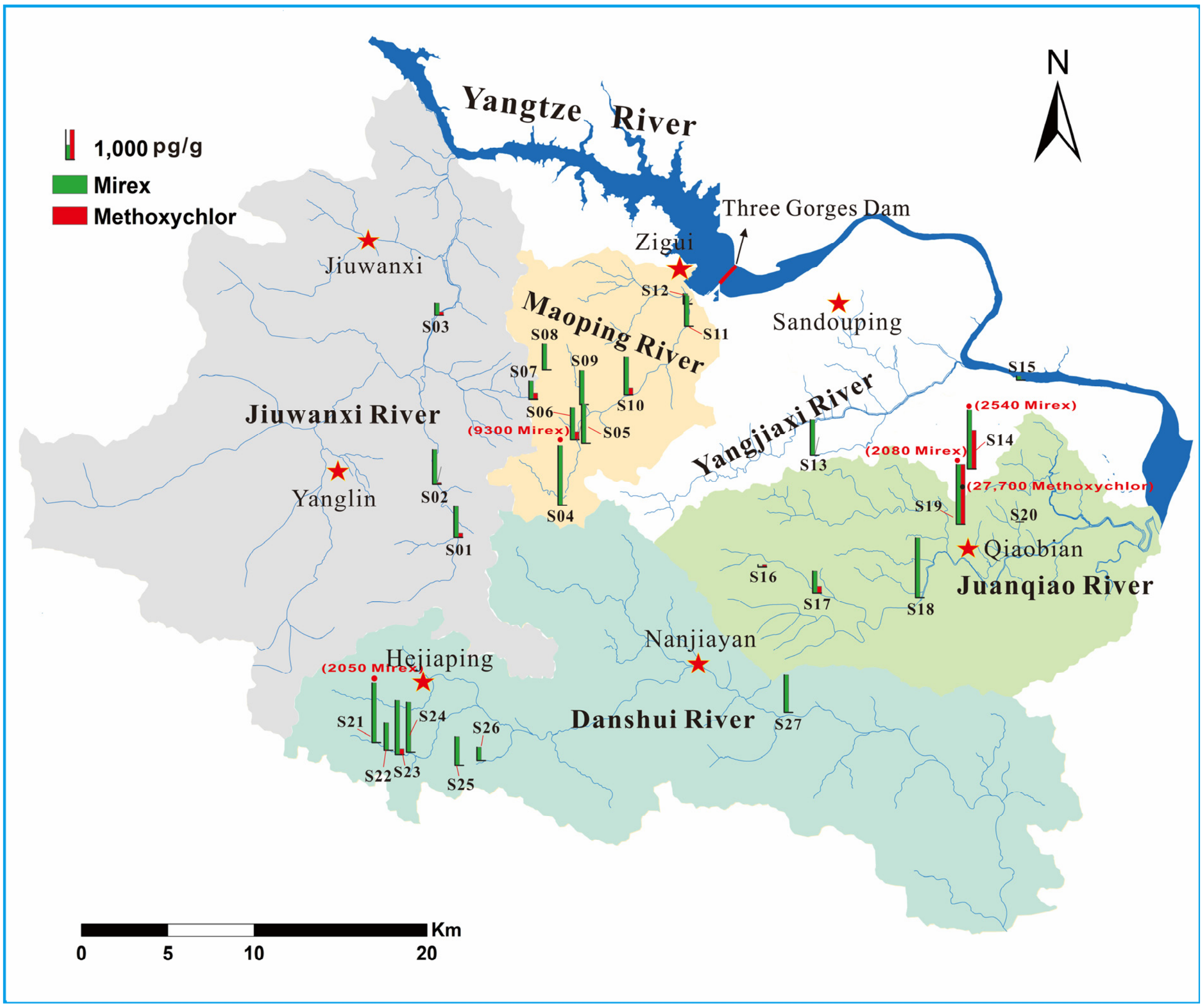

Figure S3. Spatial distributions of Mirex and Methoxychlor in different river basins.

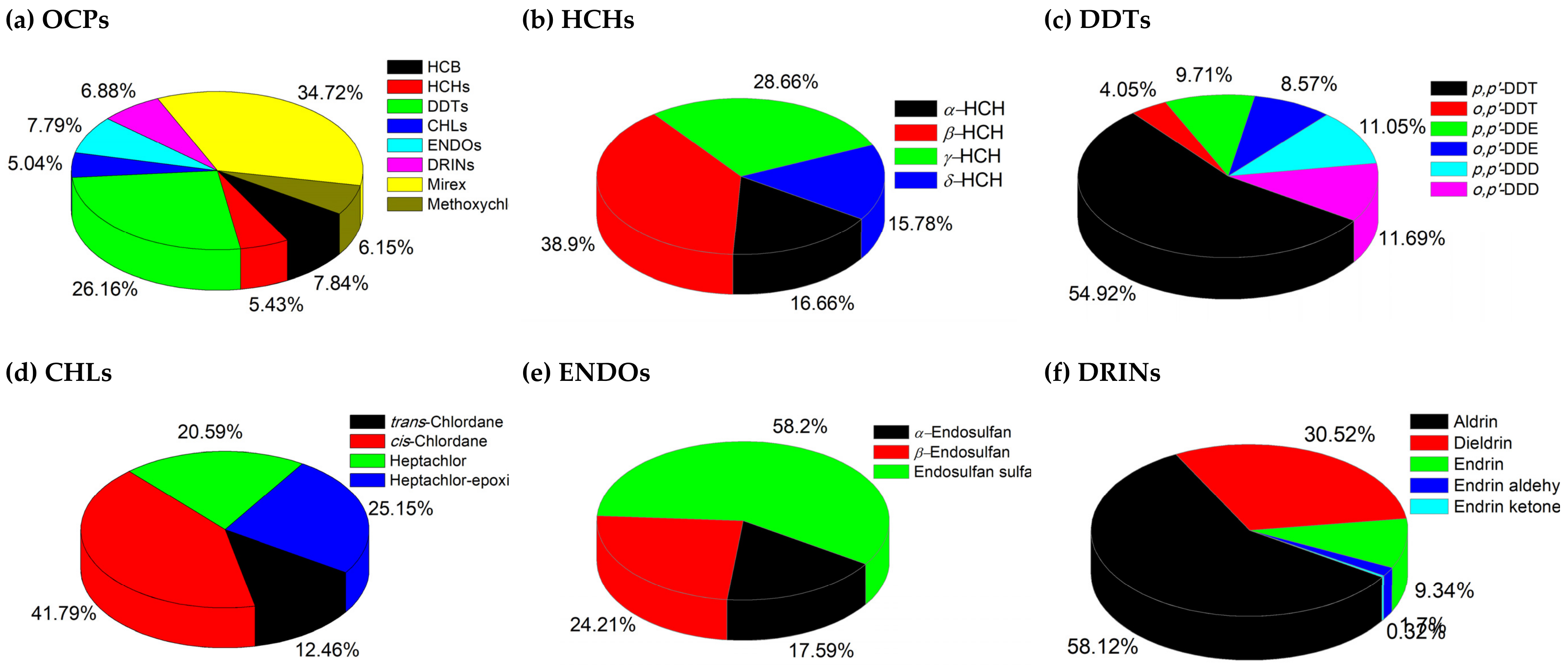

Figure S4. The OCP compositions in the soil from the study karst area.

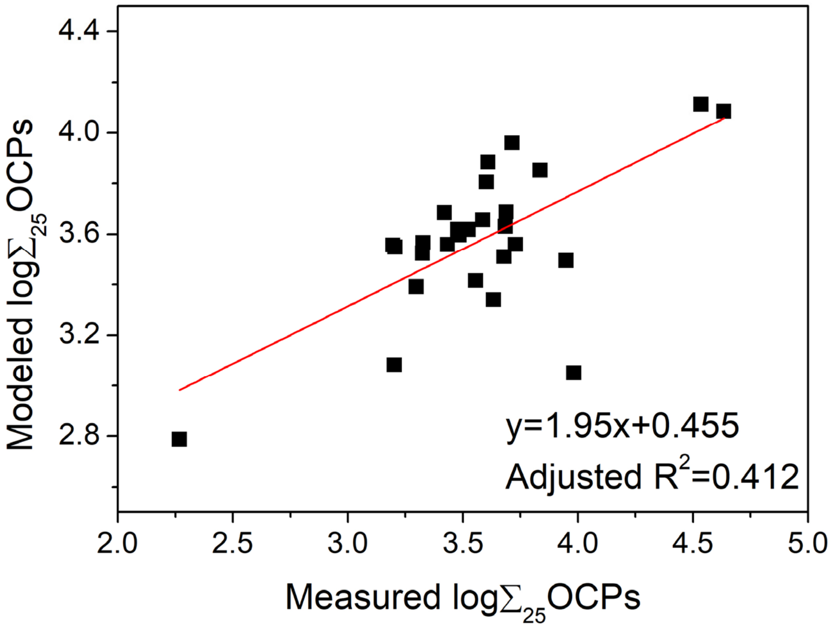

Figure S5. The linear fit between the measured log∑25OCPs and modeled log∑25OCPs by the MLRA.

References

1. Larsen, R.K.; Baker, J.E. Source Apportionment of Polycyclic Aromatic Hydrocarbons in the Urban Atmosphere: A Comparison of Three Methods. *Environ. Sci. Technol.* **2003**, *37*, 1873–1881, doi:10.1021/es0206184.
2. U.S. EPA *Exposure Factors Handbook (1997, Final Report)*; U.S. Environmental Protection Agency, Washington, DC, EPA/600/P-95/002F a-c, 1997;
3. Sultana, J.; Syed, J.H.; Mahmood, A.; Ali, U.; Rehman, M.Y.A.; Malik, R.N.; Li, J.; Zhang, G. Investigation of Organochlorine Pesticides from the Indus Basin, Pakistan: Sources, Air–Soil Exchange Fluxes and Risk Assessment. *Science of The Total Environment* **2014**, *497–498*, 113–122, doi:10.1016/j.scitotenv.2014.07.066.
4. U.S. EPA Regional Screening Level (RSL) Summary Table (TR=1E-06, HQ=0.1) 2019.
5. National Physique Monitoring Center, PRC National Physique and Health Database Available online: http://cnphd.bmicc.cn/chs/cn/analysis.php (accessed on 26 January 2021).
6. U.S. DOE The Risk Assessment Information System Available online: https://rais.ornl.gov/ (accessed on 26 January 2021).
7. Bill, B.; Juan, V.; Vishwanathan, A. Stochastic Risk Assessment of Sites Contaminated by Hazardous Wastes. *Journal of Environmental Engineering* **1998**, *124*, 380–388, doi:10.1061/(ASCE)0733-9372(1998)124:4(380).
8. NBS, PRC Average life expectancy Available online: https://data.stats.gov.cn (accessed on 26 January 2021).
9. Finley, B.; Proctor, D.; Scott, P.; Harrington, N.; Paustenbach, D.; Price, P. Recommended Distributions for Exposure Factors Frequently Used in Health Risk Assessment. *Risk Analysis* **1994**, *14*, 533–553.

10. Ministry of Housing, Spatial Planning and Environmental Management (Netherlands) Soil Remediation Circular 2009 2009.
11. State Administration for Market Regulation, Ministry of Ecology and Environment, PRC Soil Environmental Quality–Risk Control Standard for Soil Contamination of Agricultural Land (GB 15618-2018) 2018.
12. Chen, L.; Feng, Q.; He, Q.; Huang, Y.; Zhang, Y.; Jiang, G.; Zhao, W.; Gao, B.; Lin, K.; Xu, Z. Sources, Atmospheric Transport and Deposition Mechanism of Organochlorine Pesticides in Soils of the Tibetan Plateau. *Science of The Total Environment* **2017**, *577*, 405–412, doi:10.1016/j.scitotenv.2016.10.227.
13. Li, J.; Zhang, G.; Qi, S.; Li, X.; Peng, X. Concentrations, Enantiomeric Compositions, and Sources of HCH, DDT and Chlordane in Soils from the Pearl River Delta, South China. *Science of The Total Environment* **2006**, *372*, 215–224, doi:10.1016/j.scitotenv.2006.09.023.
14. Huang, H.; Ding, Y.; Chen, W.; Zhang, Y.; Chen, W.; Chen, Y.; Mao, Y.; Qi, S. Two-Way Long-Range Atmospheric Transport of Organochlorine Pesticides (OCPs) between the Yellow River Source and the Sichuan Basin, Western China. *Science of The Total Environment* **2019**, *651*, 3230–3240, doi:10.1016/j.scitotenv.2018.10.133.
15. Manz, M.; Wenzel, K.D.; Dietze, U.; Schüürmann, G. Persistent Organic Pollutants in Agricultural Soils of Central Germany. *Science of The Total Environment* **2001**, *277*, 187–198, doi:10.1016/S0048-9697(00)00877-9.
